# THE ART, WELLNESS, AND VITAL INVOLVEMENT IN AGING PROGRAM: MY WORK WITH HELEN KIVNICK

**DOI:** 10.1093/geroni/igac059.1485

**Published:** 2022-12-20

**Authors:** Linda Davis

**Affiliations:** Aging, Wellness and the Arts, INDIANAPOLIS, Indiana, United States

## Abstract

Linda Davis will discuss her partnership with Helen Kivnick on The Art, Wellness and Vital Involvement in Aging (AWVIA) Program. AWVIA began in 2009 as an innovative approach to health programming in HUD Affordable Senior Housing, employing engaging creative activities to attempt to address the despair and loneliness often found in older adults who have low social engagement, due to health, mobility, or economic circumstances. In 2013, Davis teamed up with Helen Kivnick to develop the Vital Involvement Model, which employs creative activities to increase meaningful engagement with the world. Over 6 years, AWVIA was employed by 84 HUD Senior Housing communities across the U.S. Kivnick and Davis received funding from NEA for a quasi-experimental study on the participants. Information from more than 2,000 qualitative narratives suggests the program decreased isolation, increased self-reliance, generativity, and a sense of mastery in participants. Examples of the narratives will be shared.

